# Acute Exacerbation in Interstitial Lung Disease

**DOI:** 10.3389/fmed.2017.00176

**Published:** 2017-10-23

**Authors:** Gabriela Leuschner, Jürgen Behr

**Affiliations:** ^1^Department of Internal Medicine V, Ludwig Maximilians University, Comprehensive Pneumology Center (CPC-M), German Center for Lung Research (DZL), Munich, Germany; ^2^Asklepios Fachkliniken München-Gauting, Gauting, Germany

**Keywords:** acute exacerbation, interstitial lung disease, idiopathic pulmonary fibrosis, definition, diagnosis, management

## Abstract

Acute exacerbation of idiopathic pulmonary fibrosis (AE-IPF) has been defined as an acute, clinically significant deterioration that develops within less than 1 month without obvious clinical cause like fluid overload, left heart failure, or pulmonary embolism. Pathophysiologically, damage of the alveoli is the predominant feature of AE-IPF which manifests histopathologically as diffuse alveolar damage and radiologically as diffuse, bilateral ground-glass opacification on high-resolution computed tomography. A growing body of literature now focuses on acute exacerbations of interstitial lung disease (AE-ILD) other than idiopathic pulmonary fibrosis. Based on a shared pathophysiology it is generally accepted that AE-ILD can affect all patients with interstitial lung disease (ILD) but apparently occurs more frequently in patients with an underlying usual interstitial pneumonia pattern. The etiology of AE-ILD is not fully understood, but there are distinct risk factors and triggers like infection, mechanical stress, and microaspiration. In general, AE-ILD has a poor prognosis and is associated with a high mortality within 6–12 months. Although there is a lack of evidence based data, in clinical practice, AE-ILD is often treated with a high dose corticosteroid therapy and antibiotics. This article aims to provide a summary of the clinical features, diagnosis, management, and prognosis of AE-ILD as well as an update on the current developments in the field.

## Introduction

Interstitial lung diseases (ILD) are a heterogeneous group of diseases. Despite various types of clinical presentation, disease progression, and prognosis, the common feature in most ILDs is a fibrotic destruction of the lung parenchyma. Within the clinical course of ILD, an acute exacerbation [acute exacerbations of interstitial lung disease (AE-ILD)] can occur at any time and is associated with significant morbidity and mortality (1–5). Initially, AE-ILD was described in idiopathic pulmonary fibrosis (IPF), and according to the official American Thoracic Society/European Respiratory Society/Japanese Respiratory Society/Latin American Thoracic Society IPF guideline, an acute exacerbation of idiopathic pulmonary fibrosis (AE-IPF) has been defined as an acute clinical worsening of dyspnea which develops within less than 1 month without an alternative etiology (6).

Pathophysiologically, AE-ILD resembles an acute lung injury (ALI), which presents histopathologically as diffuse alveolar damage (DAD) in most cases (7). However, DAD is not only found in autopsy studies of patients with IPF but also in patients with connective tissue-related ILD (CTD-ILD), idiopathic fibrotic non-specific interstitial pneumonia (NSIP), and chronic hypersensitivity pneumonitis (HP) (8, 9). Besides the histopathological DAD, AE-ILD and ALI have more clinical features in common, such as an increased oxygen requirement and new bilateral infiltrates on high-resolution computed tomography (HRCT) (e.g., ground-glass opacification/consolidation) (10–12).

While AE-IPF is increasingly recognized better and is perceived as a severe event with high mortality, there is only a limited amount of clinical data on AE-ILD in non-IPF ILD. The aim of this review is to provide a summary of the definition, clinical features, diagnosis, prognosis, and management of AE-ILD. Furthermore, this review will update the current developments in the field of AE-ILD not only in IPF but also in non-IPF ILD.

## Definition

Especially in IPF, great efforts have been made to establish a clear definition and diagnosis criteria for AE-IPF (6, 10, 13). In 2007, the IPF Clinical Trials Network (IPFnet) described the clinical presentation, radiological and histopathological findings of AE-IPF and developed diagnostic criteria based on the published literature (13). Just recently, an international working group revised an update on the definition of AE-IPF (Table 1) (10). In this document, AE-IPF is defined by a clinically significant respiratory deterioration developing within typically less than 1 month, accompanied by new radiologic abnormalities on HRCT such as diffuse, bilateral ground-glass opacification, and the absence of other obvious clinical causes like fluid overload, left heart failure, or pulmonary embolism (10). In contrast to the previous definition, the authors promote discrimination between a triggered AE-IPF (e.g., infection, post-procedural/postoperative or drug toxicity) and an idiopathic AE-IPF, where no trigger is identified (10). The revised definition aims to be broader and thus allow more inclusion possibilities. In the event of a clinical deterioration with unknown cause, where the criteria for AE-IPF are not met, the term “suspected AE-IPF” can be used (10). This might be the case if there are only unilateral ground glass abnormalities on HRCT or if HRCT data are even missing (13).

**Table 1:** Revised and previous definitions and diagnostic criteria for AE-IPF.

Diagnosis of AE-IPF	Revised diagnosis	Previous diagnosis
**Definition**		
	An acute, clinically significant, respiratory deterioration characterized by evidence of new widespread alveolar abnormalities	An acute, clinically significant, respiratory deteriorartion of unidentifiable cause

**Diagnostic criteria**		
– Previous diagnosis	Previous or concurrent diagnosis of IPF	Previous or concurrent diagnosis of IPF
– Clinical presentation	Acute worsening or development of dyspnea typically of less than 1 month	Unexplained worsening or development of dyspnea within 30 days
– Computed tomography findings	New bilateral ground-glass opacity and/or consolidation superimposed on a background pattern consistent with usual interstitial pneumonia (UIP) pattern	New bilateral ground-glass abnormality and/or consolidation superimposed on a background reticular or honeycomb pattern consistent with UIP pattern
– Exclusion of differential diagnosis	Deterioration not fully explained by cardiac failure or fluid overload	Exclusion of alternative causes, including left heart failure, pulmonary embolism and an identifiable cause of acute lung injury
– Concomitant Infection		No evidence of pulmonary infection by endotracheal aspirate or bronchoalveolar lavage

*AEIPF, acute exacerbation of idiopathic pulmonary fibrosis; IPF, idiopathic pulmonary fibrosis*.

## Clincal Features and Diagnostic Evaluation

Unfortunately, so far, there is no existing official definition of AE-ILD in non-IPF ILD. Since an AE-ILD in non-IPF patients resembles AE-IPF (14–16), in the clinical setting it might be reasonable to apply the definition of AE-IPF to all AE-ILD. Still, it should be pointed out, that the current definition of AE-IPF refers exclusively to IPF and that the authors of the working group report decided against a definition including other ILD (10).

The clinical presentation of AE-ILD is usually a rapid worsening of respiratory symptoms with increased dyspnea within less than 1 month (10, 13). Additional findings can be cough, increased sputum production, fever, and flu-like symptoms (1, 14, 15, 17). Since many patients present with a severe hypoxemia in the arterial blood gas analysis and respiratory failure, admission to the intensive care unit and assisted ventilation is often required (13). Established criteria for a presenting abnormal gas exchange is a Pa_O2_/Fi_O2_ ratio <225 or a decrease in Pa_O2_ of ≥10 mmHg over time (13).

**Figure 1:**
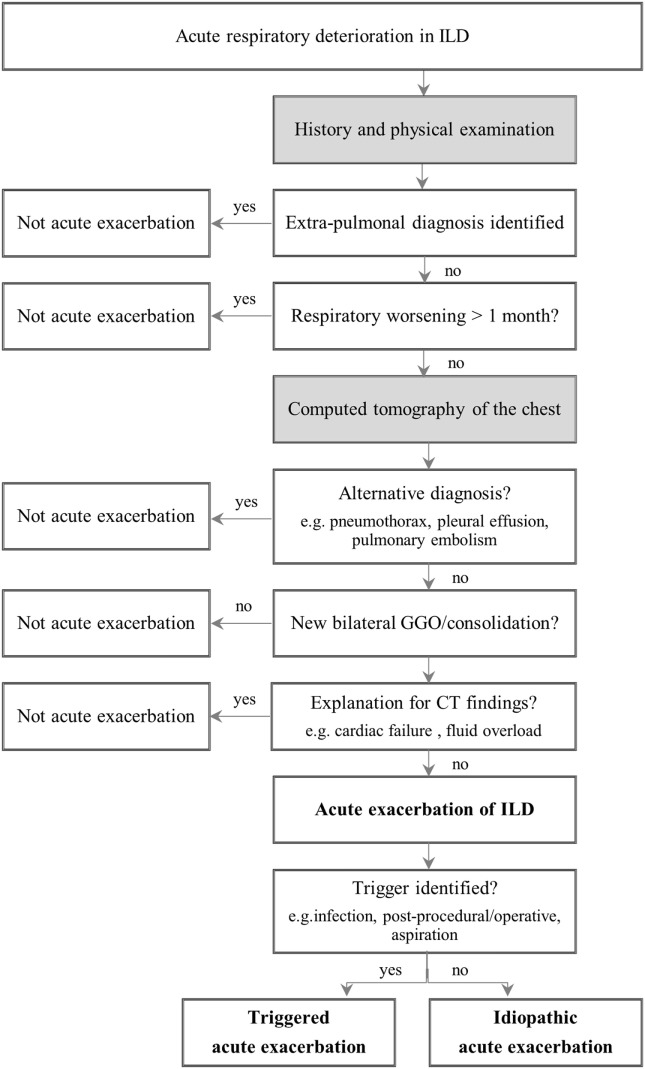
Diagnostic approach to acute exacerbation in interstitial lung disease. Adapted from Ref. (10). Abbreviation: ILD, interstitial lung disease; GGO, ground glass opacity; CT, computed tomography.

**Figure 2:**
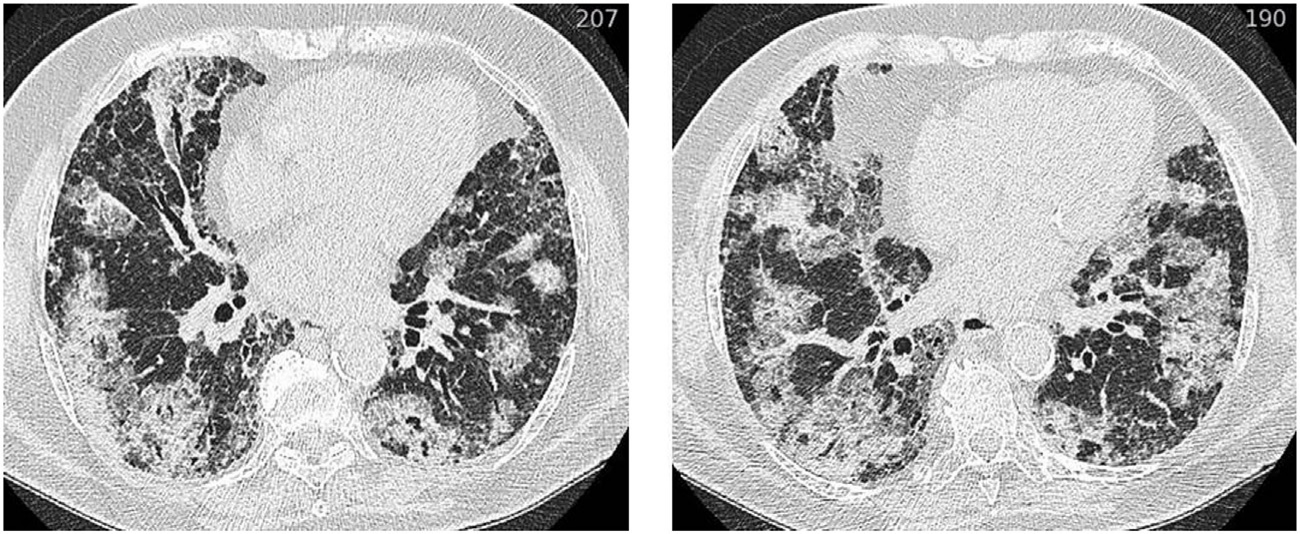
HRCT of an acute exacerbation in IPF. Axial HRCT of a patient with IPF at the time of an acute exacerbation shows extensive bilateral ground-glass opacification. Abbreviation: HRCT, high resolution computed tomography; IPF, idiopathic pulmonary fibrosis.

Still, establishing the diagnosis of AE-ILD often comprises a challenge. In order to get to this diagnosis, various diagnostic tests should be performed and differential diagnosis like myocardial infarction, pulmonary embolism, or fluid overload need to be excluded (Figure 1). An elementary part of the diagnosis is the HRCT, which should be carried out in all patients who are clinically stable. The important finding in AE-ILD is newly developed, bilateral alveolar infiltrates like ground-glass opacification with or without consolidation on HRCT (Figure 2) (6, 14, 16). Three suggested HRCT abnormality patterns are peripheral, multifocal and diffuse ground glass, with the latter two being associated with histologically DAD (13, 18). Several studies have shown that the extent of disease on HRCT seems to be related with the clinical outcome (1, 19–21). If there is no previous HRCT scan available, bilateral ground-glass opacity and/or consolidation on a background of usual interstitial pneumonia (UIP) pattern is sufficient to confirm the radiographic diagnostic criteria of AE-IPF (10). The term “suspected AE-IPF” should be used if there are only unilateral ground glass abnormalities (13).

Histologically, most AE-ILDs are characterized by a DAD, while alternative histological appearances comprise organizing pneumonia, alveolar hemorrhage, and unspecific inflammatory changes (1, 13, 15, 22, 23). In early stages, the acute injury of the lung is characterized by an interstitial edema and hyaline membranes (10). Furthermore, type II pneumocyte hyperplasia and fibroblast foci have been reported in biopsies/autopsies as well as squamous metaplasia and honeycombing with and without hyaline membranes (24).

It has been reported, that patients with AE-ILD present with elevated inflammatory laboratory values such as increased white blood cell count, elevated values of erythrocyte sedimentation rate, and C-reactive protein and increased lactate dehydrogenase (14, 23–25). Although bronchoalveolar lavage (BAL) is not done routinely in AE-ILD, it has also been shown that AE-IPF and AE-HP are associated with an increase in neutrophils in BAL (1, 9, 17, 24–27). Rarely, lymphocytosis has been observed (23, 28), and reactive type II cells hyperplasia has been dectected on BAL (24). Furthermore, BAL is still the subject of research in terms of investigating the pathogenesis of AE and identifying possible prognostic factors.

## Epidemiology

Acute exacerbations of interstitial lung disease can occur at any time during the disease, and in some cases it can be the presenting manifestation of an ILD (1, 13, 17). The exact frequency is unknown and the reported incidence rates of AE-ILD broadly vary, most likely due to differences in definition, ILD-entity and disease severity (10, 29). Furthermore, due to incomplete clinical information, definite AE-ILD cannot be confirmed in some cases, although AE-ILD is the suspected and the most probable diagnosis (10). The impact of this relevant difference was investigated in a *post hoc* analysis of the STEP-IPF trail, where a definite AE-IPF occurred in 40 per 1,000 patient-years but combining definite and suspected AE-IPF raised the number to 200 per 1,000 patient-years (30). In a recently published central adjudication on three randomized controlled trials, only 33.2% of the investigator-reported AE-IPF met the criteria (31). A meta-analysis of six randomized-controlled clinical trials identified a weighted average of 41 AE-IPF per 1,000 patient-years (32). In the INPULSIS I and II trial, the 1-year incidence of AE-IPF in the placebo-arm was 7.6% (33).

Compared to clinical trials, retrospective studies report even higher 1-year incidences of AE-IPF, ranging from 7 to 19.1% with highest risk in advanced IPF (1, 2, 34–36). Retrospective analyses of studies from the US and Japan identified the incidences of AE-IPF in approximately 52 per 1,000 patient-years (37, 38). In a registry-based US study, the annual rate of AE in IPF was 133 per 1,000 patient-years (39).

There is much less data on the frequency of AE-ILD in non-IPF ILD compared to AE-IPF. However, the majority of studies indicate that patients with IPF are at a higher risk for developing AE compared to non-IPF ILD (40–43). The estimated 1-year incidence of AE-NSIP is reported to be 4.2%, and the estimated 1-year incidence of AE-CTD ranges from 1.25 to 3.3% (14, 16). Within CTD-ILD, AE seems to be most common in patients with rheumatoid arthritis ILD (RA-ILD) (16). Since the frequency of a UIP pattern is higher in RA-ILD compared to other ILDs, the higher number of AE-RA may be explained by the observation that a UIP pattern *per se* is associated with a higher risk of AE-ILD. Thus, in patients with CTD-ILD and RA-ILD with UIP pattern, a 1-year incidence of 5.6 and 11.1% was found, respectively (14). Furthermore, the 2-year incidence of AE-HP was 11.5% among patients with chronic HP and UIP-like lesions on surgical biopsies (9).

Moreover, ethnicity may play a role, since AE-ILD were initially observed and reported in Japan and Korea, and the literature still is dominated by reports from Asian countries (1, 14, 19, 35). However, two randomized, controlled studies did not support this observation (33, 44, 45).

## Pathogenesis and Etiology

The onset and development of an AE-ILD is unpredictable and until now, it is uncertain, whether an AE-ILD is triggered by an intrinsic factor causing a progression of the underlying disease or a response to an external factor (e.g., infection, aspiration, pulmonary emboli, mechanical stretch) or both (6, 10). Most likely, environmental and genetic factors interact individually leading to AE-IPF in only a subset of patients (13). Concerning the parallels between AE-IPF and acute respiratory distress syndrome, the IPF lung may be generally more vulnerable to intrinsic and extrinsic triggers (10). Still, further research is needed to identify the underlying causes and potential biomarkers for AE-ILD.

### Epithelial Injury

During AE-IPF, alveolar injury and loss of epithelial cell integrity may be involved leading to an increased fibrin production and remodeling (13, 46). Morphologically, this leads to neutrophilia in BAL and histopathological DAD (8, 17, 24). Neutrophilic processes are potentially transmitted *via* α-defensins, as they have been shown to be upregulated in patients with AE-IPF (47, 48). α-defensins belong to a family of antimicrobial and cytotoxic peptides contained in mammalian neutrophils (49, 50). Supporting the hypothesis of epithelial injury and proliferation during AE-IPF, a gene expression study of lung tissue detected an increased expression of cyclin A2 and α-defensins together with widespread apoptosis in lungs of patients suffering from AE-IPF in comparison to stable IPF and healthy controls (47). Furthermore, α-defensins were increased in the peripheral blood of patients with AE-IPF, suggesting a potential role as biomarker (47). In a later study including patients with idiopathic interstitial pneumonia (IIP), elevated plasma levels of α-defensins in AE-IIP compared to stable IIP were also seen, but they were not useful as biomarkers due to a lack of specificity (51). Therefore, further studies are needed to clarify the role of α-defensins as biomarker.

Moreover, it could be shown that fibrocytes, which are increased in stable IPF, are even more elevated during AE-IPF (52). Fibrocytes, CD45 and collagen-1 positive cells, are mesenchymal derived progenitor cells which can migrate into injured tissue and can differentiate to fibroblast-like cells playing a role in wound repair, tissue regeneration and pulmonary fibrosis (53, 54). Patients with fibrocytes >5% of total blood leukocytes had a significantly worse survival compared to patients with fibrocytes <5% (52).

Another theory includes the involvement of alternative, so called M2, activation of macrophages in AE-IPF. M2 macrophages play an important role in tumor progression and wound healing (55), and seem to be associated with ILD (56–58). It could be shown that the pro-inflammatory chemokines CXCL1 and Interleukin 8 (produced by classically activated macrophages) and the anti-inflammatory chemokines CC-chemokine ligand (CCL)2, CCL17, CCL18, CCL22, and Interleukin 1ra (produced by alternatively/M2 activated macrophages) were elevated in BAL of patients with AE-IPF in comparison to stable IPF (26). High CCL18 levels in BAL at baseline were further highly predictive for a future AE-IPF (26).

Further markers have been studied, including Krebs von den Lungen-6 (KL-6) and surfactant protein D, which were both identified to be elevated in AE-IPF compared to stable IPF (59) and KL-6 was also increased during the event of AE-HP (60). Furthermore, an elevated serum level of KL-6 at baseline was identified as a predictor for developing AE-ILD in both, IPF and combined pulmonary fibrosis and emphysema (34, 61). KL-6 is a mucin-like glycoprotein, which is mainly expressed in type II penumocytes and bronchial epithelial cells (62). In ILD, a high expression of KL-6 has been detected in regenerating type II cells likely being the primary source of serum KL-6 (63).

Increased levels of Interleukin 6 and Interleukin 8 were also detected in patients with AE-IPF and an increase in either of them was identified to be associated with worse outcome (59, 64). Total protein C, thrombomodulin, plasminogen activator inhibitor 1 (59), and leptin (65) have also been shown to be elevated in the serum of patients with AE-IPF in comparison to stable IPF. In chronic HP, patients with elevated levels of KL-6 and surfactant protein D as well as increased neutrophils in the BAL fluid also have a higher risk for developing AE-HP.

### Autoimmunity

Heat shock protein (HSP) 47 is a human collagen-specific molecular chaperone, which is involved in the early stages of biosynthesis and secretion of collagen molecules (66). In AE-IPF, it has been shown that HSP47 serum levels were significantly higher in comparison to stable IPF (67). Furthermore, immunohistochemical analysis detected more HSP47 expression in DAD than in UIP tissues (67). Interestingly, another study identified anti-HSP70 IgG autoantibodies in 25% of patients with IPF and anti-HSP70 positivity was associated with a higher mortality and risk for AE-IPF (68). Mortality among patients with positive anti-HSP70 antibodies was significantly higher compared to patients with negative antibodies (68).

Supporting autoimmune involvement in AE-IPF, one study identified annexin 1 as an autoantigen which increased antibody production and T cell response in AE-IPF with the N-terminus of annexin 1 potentially playing a role in the pathogensis of AE-IPF (69).

### Infection

There is an increasing number of findings indicating that infection, both viral and bacterial, might be involved in some cases of AE-ILD. First, in a minority of patients with IIP suffering from AE-IIP, viral ribonucleic acid or a rise in specific immunoglobulins was detected by polymerase chain reaction or pan-viral microarray (70–74). Moreover, changes in the respiratory microbiome were recently identified showing an increased bacterial burden in BAL during an AE-IPF (27). Patients with AE-IPF experienced a markable change in the respiratory microbiome with an increase in *Campylobacter* sp. and *Stenotrophomonas* sp., as well as a significant decrease in *Veillonella* sp. compared to stable IPF (27). The hypothesis of an underlying infection is supported by the fact, that AE-IPF occurs more often between December and May (30, 75) and in the majority of studies an immunosuppressive therapy increases the risk for developing AE-IPF (30, 37, 76).

### Microaspiration

Microaspiration might also have a connection to the development of AE-ILD (10). In a *post hoc* analysis of the placebo-treated IPF patients in three clinical trials, none of the patients developing AE-IPF was on an anti-acid therapy (77). Furthermore, patients with AE-IPF had significantly higher levels of pepsin in BAL compared to stable controls, suggesting an involvement of occult aspiration (78).

## Risk Factors

Several clinical risk factors are discussed playing a potentially crucial role in developing an AE-IPF. First of all, a functionally and clinically advanced stage of disease appears to be an important risk factor. In this context, a low forced vital capacity (FVC) seemed to be the most stable risk factor (2, 26, 30, 34, 35). Other clinical risk factors include a recent decline in FVC (35, 38, 79), a low diffusing capacity of the lung for carbon monoxide (DLCO) (9, 26, 30), a low total lung capacity (9), a low 6-min walking distance (30), an impaired baseline oxygenation (30, 38), an increased dyspnea (30, 35), and a previous AE-IPF (37, 79). However, it should also be considered, that the apparent association between advanced IPF and risk of AE-IPF may be biased by the fact that in advanced disease an AE may have more obvious clinical consequences, while it may even be overlooked in less advanced disease.

In 2007, Selman et al. discriminated IPF patients as rapid or slow progressors based on the duration of symptoms before first presentation (80). Although this study did not analyze AE-IPF, the authors stated that the rapid progression of AE-IPF does not correspond to an AE-IPF (80). Still, some links might be present as the rapid progressors showed a higher rate of fibroblast migration than slow progressors and survival was significantly reduced (80). Until now there is no proof for the theory that rapid progressors and AE-IPF could have a connection but obviously, there is not enough data in this field. Given the association between a recent decline of FVC and an increased risk of AE-IPF (35, 38, 79), it would be further interesting to acknowledge daily variability of FVC. In this context, daily home spirometry is a promising clinical tool to follow the clinical course of IPF patients more closely and potentially detect AE-IPF earlier (81).

Although AE-ILD can occur in different histological forms of ILD, UIP-like lesions were identified to be associated with a higher risk for AE-ILD in patients with chronic HP and CTD-ILD (9, 14). Additional risk factors including male gender (15), a co-existing pulmonary hypertension (36), coronary artery disease (30), a higher body-mass-index (35), and exposure to increased ozone and nitrogen dioxide levels (37) have been reported. Some studies observed a higher risk in former smokers (9, 34), but this finding is inconsistent (2, 26, 82). Similarly, there are different findings concerning age as a potential risk factor: in IPF, younger patients seem to be at a higher risk for AE-IPF (26), whereas a study on CTD-ILD identified higher age as a risk factor for developing AE-CTD (16).

In a retrospective study on patients with ILD and lung cancer undergoing chemotherapy, 21.9% of the patients experienced AE-ILD during the time from diagnosis to the end of the chemotherapy treatment period (83). The authors suggested tegafur–gimeracil–oteracil potassium (S-1) and etoposide as relatively safe options in these patients. Moreover, there is one case-report about a patient with primary lung cancer and subclinical IPF, developing an AE-IPF after hypofractionated stereotactic radiotherapy (84).

A surgical biopsy is another important risk factor triggering AE-ILD. Whereas the incidence rates of developing an AE-ILD after a surgical biopsy for ILD-diagnosis finding are reported to be less than 2.5% (40, 41, 43, 85), AE-ILD after pulmonary resection due to lung cancer can occur in 3–32% (40, 86–88). A decreased FVC and DLCO seemed to be additional risk factors for patients with ILD developing a respiratory deterioration after lung surgery (43, 89). Pulmonary surgery itself seems to be a risk factor, but AE-ILD has also been reported in non-pulmonary surgery and throughout major surgeries the incidences was 3.3% in a study from Korea (90, 91). In IPF, there might be an association between AE-IPF and BAL, as few, individual cases of AE-IPF following BAL exist (92, 93). Furthermore, as this will become increasingly important in the future, the risk of AE-ILD after cryobiopsy needs to be investigated. However, data in this field is still limited and so far, only single cases of AE-ILD following cyrobiopsy have been reported (94, 95).

## Prognosis

Acute exacerbations of interstitial lung disease is a life-threatening event and the mortality rate is high. It is assumed that between 35 and 46% of deaths in IPF are caused by AE-IPF (35, 96, 97). In a large number of studies, the in-hospital mortality in AE-IPF is estimated over 50% (1–4, 17, 19, 20, 36, 70, 98) and the median survival after AE-IPF is between 1 and 4 months (2, 36, 75, 82). In IPF, the 1-month mortality ranges between 37 and 53% (37, 99), and the 3-month mortality rate ranges from 63.8 to 73.7% (5, 19, 99). The existing data suggest that patients with IPF have a worse survival compared to ILD patients other than IPF; nonetheless, AE-ILD is also fatal in non-IPF ILD (5, 74). In a study including IPF and non-IPF patients, the overall survival after admission for AE-ILD was 67% at 1 month and 40% at 3 months (4). Similarly to IPF, the highest overall mortality rate of AE-ILD is seen in AE-HP (75–100% mortality) (9, 15). Mortality of AE-ILD in other ILDs ranges from 34 to 83% (5, 16).

Some potential prognostic factors have been identified. First of all, lower baseline pulmonary function parameters (FVC and DLCO) as well as a more impaired oxygenation are associated with a worse outcome in AE-IPF (2, 35, 75). Furthermore, a higher fibrosis score or more extensive disease on HRCT seems to be of prognostic relevance (1, 19–21). A lymphocytosis >15% in the BAL might be another prognostic factor for a favorable outcome in patients with AE-IIP (28). Several markers in the blood could also be potential prognostic markers including lactate dehydrogenase (9, 19, 75), C-reactive protein (2), KL-6 (19, 21), circulating fibrocytes (52), and anti-HSP70 autoantibodies (68). Just recently, Kishaba et al. developed a staging system for AE-IPF, which includes some of these prognostic factors (19).

## Treatment

So far, there is a lack of evidence based data on effective therapies in AE-ILD. In clinical practice, AE-ILD is often treated with high-dose systemic corticosteroid therapy and antibiotics (9, 14, 16). In AE-IPF, the current international guidelines give a weak recommendation on the treatment with corticosteroids emphasizing that this recommendation is based on anecdotal reports of benefit and the high overall mortality in AE-IPF (6). The authors further point out, that there was a consensus to promote supportive care as an important therapy strategy (6). This includes palliation of symptoms, e.g., with opioids, and supply of oxygen in hypoxemia. Still, there are different opinions on the length of supportive care, and regarding the use of mechanical ventilation (10). Based on an estimated 90% in-hospital mortality, the international guidelines on the management of IPF make a weak recommendation against the use of mechanical ventilation in the case of respiratory failure due to the underlying lung disease (6). The authors point out that this decision has to be made case-by-case together with the physician, the patient, and the family and in accordance with the individual goals of care (6). As a bridge to lung transplantation, mechanical ventilation, or extra-corporal membrane oxygenation may be appropriate and successful in selected patients (6, 100). In a recent retrospective cohort study, the mortality rate in IPF patients undergoing mechanical ventilation significantly decreased from 58.4% in 2006 to 49.3% in 2012 (101). This reminds us to carefully analyze every single patient before a decision for or against mechanical ventilation is taken.

Several studies on different therapy regimens in AE-IPF, other than high-dose intravenous corticosteroids mono, have been published (Table 2). In smaller, observational studies, it could be shown that the combination of a steroid-pulse therapy with oral tacrolimus (82) or cyclosporine (102–104) was superior to the corticosteroid mono therapy in terms of prognosis in IPF. Other studies identified a positive effect of a treatment with rituximab with plasma exchange and intravenous immunoglobulin (105), polymyxin B-immoblilized fiber column perfusion (99, 106–109), and intra-venous thrombomodulin (110–114). Still, the benefits seen in these studies have to be critically assessed since these were all observational studies with either a historical control or a parallel, untreated control arm, potentially excluding very ill patients from the experimental arm (10). One randomized trial investigated the benefit of a procalcitonin-guided antibiotic therapy compared to a clinical-driven antibiotic therapy but no difference in mechanical ventilation and mortality was seen (115). Several studies reported about a combination therapy of corticosteroids with other immunosuppressant drugs like cyclophosphamide, but it remains unclear whether this is beneficial (20, 21, 116).

**Table 2:** Medical treatment of AE-IPF other than high-dose intravenous corticosteroids mono therapy.

Treatment	Reference	Study design	Number of patients	Treatment/intervention	Clinical outcome
Tacrolimus	Horita et al. (82)	Single-center, retrospective study	15	Steroids mono versus combination steroids plus tacrolimus	Significantly better survival in tacrolimus-group

Cyclosporine	Inase et al. (102)	Single-center, retrospective study	14	Steroids mono versus steroids followed by cyclosporine	Cyclosporine seemed to prevent re-exacerbation and improve survival (no data on significance level)
	Homma et al. (103)	Retrospective study	44	Effect of treatment with steroids mono versus steroids plus cyclosporine before AE-IPF	Significantly better survival in cyclosporine-group
	Sakamoto et al. (104)	Single-center, retrospective study	22	Steroids mono versus combination of steroids plus cyclosporine	Significantly better survival in cyclosporine-group

Rituximab	Donahoe et al. (105)	Pilot- phase I/II-study; historical controls	31	Steroids mono versus combination of steroids plus rituximab/therapeutic plasma exchanges and IVIG in severely ill IPF	Significantly better 1-year survival in rituximab group

PMX	Seo et al. (99)	Open-label pilot trial	6	Combination of steroids plus PMX	Potential beneficial effect of treatment with PMX
	Abe et al. (106)	Multi-center, retrospective study	160	Combination of steroids plus PMX	PMX improved oxygenation and may improve survival in IP patients with AE
	Abe et al. (107)	Single-center, retrospective study	45	Steroids mono versus combination of steroids plus PMX	PMX treatment significantly improved oxygenation
	Oishi et al. (108)	Single-center, retrospective study	50	Steroids mono versus combination of steroids plus PMX	Significantly better 1-year survival in PMX group
	Oishi et al. (109)	Single-center, retrospective study	26	Stable IPF and healthy controls versus combination of steroids plus PMX in AE-IPF	PMX treatment significantly improved oxygenation

Thrombomodulin i.v.	Isshiki et al. (110)	Single-center, retrospective study	41	Steroids mono versus combination of steroids plus recombinant human soluble thrombomodulin	Thrombomodulin treatment significantly improved 3-month survival
	Kataoka et al. (112)	Single-center, retrospective study	40	Combination of steroids and cyclosporine versus combination of steroids and cyclosporin plus recombinant human soluble thrombomodulin	Thrombomodulin treatment significantly improved 3-month survival
	Tsushima et al. (111)	Single-center, combined prospective and retrospective study	20	Combination of steroids plus recombinant human soluble thrombomodulin	Thrombomodulin treatment significantly improved oxygenation
	Hayakawa et al. (113)	Single arm, non-randomized prospective clinical trial; historical controls	23	Steroids mono versus combination of steroids plus recombinant human soluble thrombomodulin	Thrombomodulin plus steroid pulse therapy improved oxygenation and may improve overall survival
	Abe et al. (114)	Single-center, prospective, non-randomized study	22	Steroids mono versus combination of steroids plus recombinant human soluble thrombomodulin	Thrombomodulin treatment significantly improved 3-month survival

Procalcitonin-guided antibiotic therapy	Ding et al. (115)	Single-center, prospective, randomized study	68	Clinically guided versus procalcitonin-guided antibiotic therapy	Procalcitonin-guided antibiotic therapy had no benefits on survival

Cyclophosphamide	Akira et at (20)	Single-center, retrospective study	58	Steroids mono and combination of steroids mono plus cyclophosphamid	No data on treatment-related outcome
	Fujimoto et al. (21)	Multi-institutional, retrospective study	60	Steroids plus cyclophosphamide and steroids plus cyclosporine	No data on treatment-related outcome
	Yokoyama et al. (116)	Single-center, retrospective study	11	Steroids mono and combination of steroids mono plus cyclophosphamide and combination of steroids mono plus cyclosporine	No data on treatment-related outcome

*AE-IPF, acute exacerbation of idiopathic pulmonary fibrosis; IPF, idiopathic pulmonary fibrosis; i.v., intravenous; IVIG, intravenous immunoglobulin; PMX, polymyxin B-immobilized fiber column*.

Currently, there are indications that an anti-acid therapy could have a protective effect against AE-IPF, as in an analysis of patients from the placebo arm of three large clinical trials, an anti-acid therapy was reported to have a potentially preventive effect on the development of AE-IPF (77). The same potentially preventive effect applies for antifibrotic drugs, although, so far, there is no sufficient data on whether an antifibrotic therapy with nintedanib or pirfenidone should be paused or continued in the event of an AE-IPF. In a phase II trial, patients receiving pirfenidone had a significant reduction in AE-IPF compared to placebo (117). However, a subsequent phase II trial could not reproduce this finding (44). After AE-IPF was not included as an endpoint in the three phase III trials ASCEND and CAPACITY (118, 119), a pooled analysis recently showed that patients receiving pirfenidone had a lower risk for respiratory-related hospitalization compared to healthy controls (120). Interestingly, there is data that the perioperative use of pirfenidone might prevent postoperative AE-IPF (121). In contrast to pirfenidone, AE-IPF was consequently included in the nintedanib clinical study program as a key secondary endpoint; however, the role of nintedanib on AE-IPF needs to be fully understood. Whereas the phase II trial of nintedanib identified a delay in time to the first investigator-reported AE in the nintedanib arm (122), only one of the two INPULSIS phase III twin-trials showed a significant effect of nintedanib on AE-IPF (33). Pooled analysis of the data showed a highly significant prolongation of the time to first AE in IPF due to treatment with nintedanib, thus confirming its preventive effect (45, 123). Still, at this point, more data is needed to validate the effect of pirfenidone and nintedanib in AE-ILD.

Apart from these potentially effective therapeutic approaches, there are a number of drugs that seem to have no preventive effect on AE-IPF, including acetylcystein mono therapy (124), sildenafil (125), bosentan (126), interferon-gamma 1b (127), warfarin (128), ambrisentan (129), and imatinib (130). A combination “triple” therapy (prednisone, azathioprine and acetylcysteine), might even increase the risk for developing AE-IPF (131).

As in AE-IPF, AE-ILD in non-IPF ILD is often treated with a high dose, systemic corticosteroid therapy together with broad-spectrum antibiotics (9, 14, 16). There are some studies additionally using cyclosporine A or cyclophosphamide, but there are no reports on whether there was any benefit in these therapies (9, 16). Similar to IPF, treatment with intra-venous thrombomodulin significantly improved 3-month survival in AE-NSIP (114).

In order to address all therapeutic approaches in this context, one study should be mentioned focusing on a non-steroid approach in AE-IPF: in the event of AE-IPF, prior immunosuppression was immediately stopped and patients were only treated with best supportive care and broad-spectrum antibiotics (132). The median survival of all patients was 1.73 months. Analyzing the single event of AE-IPF, 50% of AE-IPF episodes were survived. Overall, 35.3% of the patients survived AE-IPF and the 1-year survival of the survivors was 83%. Interestingly, patients who had never been treated with immunosuppressant drugs before had a significantly better survival. The 1-year survival in the “never treated” group was 65%, whereas the patients, who had a history of immunosuppression, had a 1-year survival of 17%. Unfortunately, no comparison with a high-dose steroid therapy during AE-IPF was investigated in the study. Nevertheless, this underlines again the lack of evidence based data on therapy strategies in AE-ILD and the necessity for further studies in this field.

## Future

Acute exacerbations of interstitial lung diseases are severe events with a high mortality rate. Therefore, it is important to gain further knowledge in this field. As it has been shown that in IPF, an early referral to a specialized center is crucial for survival in general (133), in AE-ILD an early diagnosis and referral might also be important for the patients’ prognosis. Therefore, effort should be made to detect early signs of AE-ILD and identify patients who are at a higher risk for developing AE-ILD.

Potential treatment options should be studied in randomized, controlled trials. The currently revised definition of AE-IPF will hopefully allow a more uniform diagnosis, which will help to conduct well designed clinical trials in IPF (10). However, it should be emphasized that, even if it largely follows the framework of AE-IPF guidelines, an official, uniform definition for AE-ILD is needed in the future.

Biomarkers, which can be obtained in an easy and harmless way, are needed to identify patients at a higher risk for developing AE-ILD before symptoms and HRCT features are present. Since biomarkers in BAL might be difficult to obtain in a severely ill patient, serum markers are particularly interesting because of their easier accessibility. Furthermore, daily home spirometry might be a potential tool to understand the clinical course of AE-PF better and even possibly detecting AE-IPF in an early stage (81). There is evidence that home spirometry can potentially improve endpoint efficacy in clinical trials of IPF-therapeutics (134). Therefore, an effort should be made to design studies in that field analyzing the benefit of daily home spirometry in patients with ILD and help establishing home spirometry in clinical, daily routine.

## Conclusion

Acute exacerbations of interstitial lung disease is a life-threatening event with a high in-hospital mortality rate. The clinical presentation of AE-ILD is similar in non-IPF and IPF, but AE-ILD in non-IPF ILD is less common and the clinical course is less fatal compared to IPF. The new working group report on AE-IPF supports that there are both, idiopathic and triggered AE (e.g., triggered by an infection) (10). So far, there is no evidence as to whether a triggered AE-ILD has a worse prognosis than an idiopathic AE-ILD. Due to the lack of evidence-based therapy options, more studies in this field are urgently needed.

## Author Contributions

GL and JB wrote the manuscript and have approved the final version of the manuscript for submission.

## Conflict of Interest Statement

GL received travel funding from Intermune and Novartis. JB received personal fees from Actelion, grants from Actelion, personal fees from Bayer, personal fees from Boehringer-Ingelheim, personal fees from Roche; JB is member of national and international IPF guideline committees.

## Abbreviations

AE-CTD, acute exacerbation of connective tissue disease related interstitial lung disease; AE-HP, acute exacerbation of chronic hypersensitivity pneumonitis; AE-IIP, acute exacerbation of idiopathic interstitial pneumonia; AE-ILD, acute exacerbations of interstitial lung disease; AE-IPF, acute exacerbation of idiopathic pulmonary fibrosis; AE-NSIP, acute exacerbation of non-specific interstitial pneumonia; AE-RA, acute exacerbation of rheumatoid arthritis and interstitial lung disease; ALI, acute lung injury; BAL, bronchoalveolar lavage; CCL, CC-chemokine ligand; CTD-ILD, connective tissue-related interstitial lung disease; DAD, diffuse alveolar damage; DLCO, diffusing capacity of the lung for carbon monoxide; FVC, forced vital capacity; HP, hypersensitivity pneumonitis; HRCT, high-resolution computed tomography; HSP, heat shock protein; IIP, idiopathic interstitial pneumonia; ILD, interstitial lung disease; IPF, idiopathic pulmonary fibrosis; KL-6, Krebs von den Lungen-6; NSIP, non-specific interstitial pneumonia; RA-ILD, interstitial lung disease in patients with rheumatoid arthritis; UIP, usual interstitial pneumonia.
